# Comparative analysis of resected prostate weight in diabetic and non-diabetic benign prostatic hyperplasia Patients

**DOI:** 10.22088/cjim.8.2.99

**Published:** 2017

**Authors:** Emadoddin Moudi, Abazar Akbarzadeh-Pasha

**Affiliations:** 1 Cancer Research Center, Health Research Institute, Babol University of Medical Sciences, Babol, Iran.; 2 Department of Urology, Shahid Beheshti Hospital, Babol University of Medical Sciences, Babol, Iran.

**Keywords:** Diabetes, Benign prostatic hyperplasia, Prostate weight, PSA

## Abstract

**Background:**

Benign prostatic hyperplasia (BPH) is the most common benign tumor in men. The etiology of BPH is still unresolved and multiple systems are likely to be involved. The effects of diabetes on urinary system are a risk factor for BPH. We then assessed the effects of diabetes on the parameters related to BPH, especially weight and volume.

**Methods:**

This study was conducted on patients with BPH who underwent surgery during 2010-2013. The patients’ demographic and clinical data including age, height, weight, history of diabetes, abdominal sonography, prostate-specific antigen(PSA), fasting blood sugar (FBS), triglyceride, and cholesterol, resected sample weight, and pathological diagnosis were extracted.

**Results:**

The mean age of all 225 patients (35 (15.6%) diabetic patients and 190 (84.4%) non-diabetic patients) who entered the study was 71.5±8.7 years. The patients were divided in to 3 body mass index (BMI) groups: 48 (21.3%) were normal, 151 (67.1%) were overweight and 26 (11.6%) were obese. The mean weight of resected prostate was higher in diabetic patients (22.9±6.9 vs 21.7±14.3, P=0.02). The resected prostate weight had a significant relationship with BMI (P=0.001), prostate–specific antigen (PSA) level (P=0.001), and prostate volume sonography (P=0.001). No significant relationship was detected between resected prostate weight with age, FBS and triglyceride however, it is significant with cholesterol.

**Conclusion:**

We concluded that diabetes has a role in the development and progression of BPH with effect on prostate weight and volume. As well, BMI is a risk factor in BPH progression.

Benign prostatic hyperplasia (BPH) is the most common benign tumor among men which is related to age. This disease has a prevalent complication worldwide and about 50% of men older than 60 years have this complication. BPH is a non-malignant enlargement or growth of the prostate gland which is characterized by the increase of epithelial and stromal cells. The etiology of BPH is not recognized yet, but possibly there are different systems (endocrine, nervous, immunity and vascular) involved in this situation by their overlap with local factors (1, 2). Aging, testosterone level, inflammation, changes in cell signaling, familial background, high BMI and diabetes are the main BPH risk factors (3). In BPH, the central sections of the prostate growth which pressure the urethra passing the prostate cause urinary problems such as, a feeling of lack of complete urinary bladder drainage, delay in urine (hesitancy), and urine frequency (4). 

On the other hand, type 2 diabetes is a prevalent endocrine disease which involves different organs such as genitourinary system. Diabetic autonomic neuropathy causes different disorders such as, cystopathy, erection disorders and sexual disorders. By exacerbation of the urinary bladder contraction, the urinary bladder capacity and the residue of the urine increase cause symptoms such as, urine hesitancy, urine incontinence and recurrent urinary infections (5, 6). High prevalence of both diseases and shared urinary symptoms caused the suspicion that both diseases are related to each other in any way. This is possible that diabetes is a risk factor or etiology of BPH or at least it may be effective on the process and complications of BPH (7). Since there is organomegaly in diabetic patients generally (5), this question is addressed whether the higher prevalence of BPH relates to the prostate volume or diabetic-related cystopathy and neuropathy. The prostate weight which is equivalent to its volume is an important feature in BPH and it can be a predictive factor of the process and symptoms. We decided to compare this element in diabetic and non-diabetic BPH patients under transurethral resection of the prostate (TURP) or open surgery and investigated its relationship to the level of fasting blood glucose level. Also, the PSA level was compared between the diabetic and non-diabetic groups. 

## Methods

From 2010 to 2013, the medical records of all patients who referred to Shahid Beheshti Hospital, Babol with symptoms of lower urinary tract symptoms (LUTS) and BPH and underwent TURP surgery or open prostatectomy were investigated. Patients’ information included age, height, weight, diabetes background, prostate volume in abdominal ultrasound, urinary residue, PSA level, type of operation, preoperative FBS level, triglyceride level and preoperative blood cholesterol and pathology diagnosis and prostate reseeted weight. According to weight and height of patients, the participants were classified to 3 BMI groups: normal (BMI<25kg/m^2^), overweight (25kg/m^2^≤ BMI<30kg/m^2^) and obese (BMI≥30kg/m^2^). The patients without report of prostate weight, prostate cancer, incomplete record and previous surgery for prostate were excluded. Data were analyzed using SPSS Version 21 and chi-square test, t-test, Man-Whitney and Pearson’s and Spearman’s correlation coefficients were utilized. A p<0.05 indicated the significance.

## Results

In this research, the records of the 300 patients who referred to Babol Shahid Beheshti Hospital due to lower urinary tract symptoms and BPH diagnosis from 2010 to 2013 and underwent the TURP or open prostatectomy were investigated. Among this group, 75 (25%) patients were excluded due to incomplete record, previous prostate interventions, any diagnosis other than BPH in pathology or lack of reporting pathology prostate weight finally, 225 patients were entered in the study. 

The mean age of the patients was 71.5±8.7 years (range 50-95 years, median 73 years). 35 (15.6%) patients with self-reported diabetes and 190 (84.4%) patients did not mention their diabetes background. Among the diabetic patients, 10 (28.5) patients received metformin and 18 (51.4%) patients took glibenclamide and 5 (14.2%) patients received both drugs. 2 (5.7%) patients used insulin to control diabetes. The mean period of diabetes was 4.6±3.2 years and the mean period of anti-diabetes drug use was 2.9±1.7 years. 208 (92.4%) patients underwent TURP while 17 (7.6%) patients had open prostatectomy. Totally, all diabetic patients had TURP. Simultaneous operations was performed on 30 (13.3%) patients among which 21 (9.3%) patients underwent litholapaxy and 9 (4%) had cystolithotomy. Age, BMI and FBS before operation, prostate estimated volume in ultrasound, PSA level and weight of resected prostate for the patients are listed in table 1. The variables of preoperative triglyceride and cholesterol levels and urinary residue were reported in some records and their mean value is reported in table 2. 

Patients were categorized into three BMI groups: 48(21.3%) were normal, 156 (67.1%) were overweight and 26 (11.6%) were obese. In total, 78.7% of patients were overweight or obese (BMI>25kg/m^2^). All diabetic patients had BMI>25kg/m^2^. Diabetic patients had resected prostate weight more than the non-diabetic patients (mean 22.9±6.9 vs. 20.7±14.36, P=0.02). In addition, the mean BMI (29.5±2.18 vs. 26.7±2.6, P=0.025), ultrasound estimated prostate volume (72±19.6 vs. 63.2±25.2m, P=0.01) and preoperative PSA level (7.6±2.8 vs. 4.4±2.2, P=0.02) were reported higher in diabetic patients than non-diabetic patients. There was no significant difference between diabetic and non-diabetic patients’ urinary residue. The resected prostate weight had correlation with BMI directly (P=0.001), PSA level and ultrasound estimated volume significantly had relationship with the resected prostate weight (P=0.001). But there was no relationship between the resected prostate weight and age, FBS, triglyceride, cholesterol level. The mean PSA level and ultrasound estimated volume were in direct relation to patients’ BMI (P=0.02 and P=0.01, respectively), but the urinary residue and BMI did not have any significant relationship (P=0.2). The patients FBS level and ultrasound estimated volume did not have any significant relation to PSA level. 

**Table1:** Age, resected prostate weight, ultrasound estimated volume, PSA level, BMI, FBS in diabetic and non-diabetic patients with BPH

**Variables**	**Non-diabetic** **n=190**	**Diabetic** **n=35**	**Total ** **n=225**	**P value**
Age(year)	71.8±8.7	70.3±8.4	71.5±8.7	0.1
Resected prostate weight(gr)	20.7±14.3	22.9±6.9	21.9±13.4	0.02
Ultrasound estimated volume(ml)	63.2±25.2	72±19.6	64.4±24.5	0.01
PSA* level(ng/ml)	4.4±3.0	7.3±9.9	5.9±3.3	0.02
BMI** (kg/m^2^)	27.7±2.6	29.5±2.1	27.2±2.7	0.025
FBS(mg/dl)	101.2±15.2	158.7±41.6	118.6±30.7	0.01

* PSA: Prostate specific antigen

** BMI: Body mass index

**Table 2:** Urinary residue, triglyceride and cholesterol level in diabetic and non-diabetic patients with BPH

**Variables**	**Total** **n=225**	**Diabetic** **n=35**	**Non-diabetic** **n=190**	**P value**
Urinary residue(ml)	213.0±126.7	198.0±119.1	218.0±128.2	0.1
Triglyceride(mg/dl)	126.3±71.2	181.5±94.7	113.0±59.5	0.02
Cholesterol (mg/dl)	180.3±40.4	216.1±39.4	171.8±36.4	0.025

## Discussion

The prevalence of diabetes among the studied population was 15.6%, while the prevalence of diabetes in patients with BPH who are candidates for surgery is 5-17% was reported in different research (8, 9). Additionally, diabetic patients had a significantly higher BMI than non-diabetic patients which are consistent with previous literatures (10). The mean PSA level in all patients was 5.9±3.3 which was higher than previous research (11-15). The reasons for the high PSA level mean are 1) remarkable number of the patients had high PSA level and negative biopsy for prostate cancer; 2) majority of patients were catheterized during the PSA evaluations which could increase the level of PSA. The results of this study indicated that the resected prostate weight via surgery intervention in BPH patients with diabetes is higher than non-diabetic patients. Past research did not report the investigation of this relationship (8, 9). Furthermore, like resected prostate weight, the abdominal ultrasound prostate size and PSA level is higher in diabetic patients than the non-diabetic patients which is consistent with Ozden and Qu’ results (11, 16). Hammarstein et al. also indicated that diabetic patients with LUTS have larger prostate than non-diabetic patients (17). Unlike the mentioned studies, Sarma et al. did not find any relationship between diabetes and prostate volume and PSA level and considered how diabetes affects more LUTS (18). In another study, aiming to investigate diabetes treatment and PBH development, they concluded that diabetes treatment does not significantly affect the prostate volume and PSA level (19). In Burke et al.’s study diabetes and BPH development, found no relationship between annual prostate volume change and PSA level with diabetes (14). Out of 225 patients in the present research, only 68 patient records reported urinary residue. This variable was not of significant difference between diabetic and non-diabetic patients which was consistent with Michel et al.’s study (20). 

In the analysis on the relationship between metabolic syndrome and BPH as a secondary goal of present research, the resected sample weight of prostate was compared to BMI, triglyceride and cholesterol level. The resected prostate weight was in direct relation with patients’ BMI, but it was not is relation with triglyceride and cholesterol levels. In an evaluation conducted by Nandeesha et al., the cholesterol levels in non-diabetic patients with BPH were higher than the normal subjects (21). Moreover, in our results, the BMI of the patients with ultrasound estimated volume and PSA level were in direct relationship. Similarly, in Kim et al.’s study, the results were consistent in which the BMI of patients with BPH was in reverse relation to PSA level and in direct relation to prostate volume on an international prostate symptom score (IPSS) (12). Besides, in Byun et al.’s study, the research on healthy individuals in terms of BPH, the patients with metabolic syndrome had higher study prostate volume and PSA level. The more metabolic syndrome criteria were seen in individuals, the larger prostate volumes and PSA level they had (14). 

In contrast, Yim et al.’s evaluation of healthy individuals younger than 50 years of age from BPH perspective concluded that individuals with metabolic syndrome do not have larger prostate (13). The relationship between FBS with resected prostate weight, or ultrasound estimated prostate volume and PSA level was investigated and a significant relationship was not found between these variables. Unlike this result, in Yim et al.’s study, the patients with abnormal FBS result had larger prostate than the patients with normal FBS result (13). Likewise, in Qu’s research, the abnormal level of FBS is related to an increase in prostate volume (11). In many past investigations, the effect of diabetes on BPH symptoms particularly IPSS was evaluated (11, 12, 20), but in the present research, due to its retrospective nature and lack of availability of symptoms of accurate process, this relationship has not been investigated. Instead, the effects of diabetes on BPH laboratory factors such as prostate weight and volume and PSA level were mostly investigated.

To accurately investigate whether diabetes as an independent factor causes the indications of surgery in patients with BPH or not, it is necessary to conduct a prospective research by following-up the patients’ symptoms in diabetic and non-diabetic groups. Further, the relationship between the elements related to metabolic syndrome with BPH has to be investigated more completely. 

In conclusion, more resected prostate weight in present research was related to the larger prostate volumes and since larger size of prostate cause more symptoms and exacerbation of the BPH process, it can be concluded that diabetes can be effective on BPH development process by affecting the prostate volume. Of course, this influence is not possibly related to the higher patients’ blood FBS level. Obesity is also one of the metabolic syndromes related to the more volume and weight prostates. Consequently, obese individuals are mostly exposed to the risk of larger prostate and as a result, the severity of symptoms is more in these individuals. The relationship between triglyceride and blood cholesterol with prostate volume and weight has not been proven in the present research. Definitely, the sample size has not been sufficient in this regard.

In the end, considering the results of the current study and other research, it can be concluded that diabetes can increase the lower urinary tract symptom exacerbation, particularly the prostate volume development. Its volume and weight increase due to its effects on both the urinary bladder and prostate. Besides, overweight individuals (high BMI) have larger prostate which indicate the effect of obesity on BPH symptoms.
